# Inhibitory mechanism of ANP on aldosterone production in human adrenocortical cells

**DOI:** 10.1530/EC-26-0107

**Published:** 2026-08-12

**Authors:** Noriko Morishima, Atsuhiro Ichihara, Daisuke Watanabe, Noriyoshi Takano, Satoshi Morimoto, Yasufumi Seki

**Affiliations:** ^1^ Department of Biobank Data Management, Tokyo Women’s Medical University, Tokyo, Japan; ^2^ Department of Endocrinology and Hypertension, Tokyo Women’s Medical University, Tokyo, Japan; ^3^ Department of Medicine, Tokyo Women’s Medical University Adachi Medical Center, Tokyo, Japan

**Keywords:** ANP, aldosterone, cyclicAMP, cyclicGMP, ANP receptors

## Abstract

**Objective:**

The aldosterone-suppressing effect of natriuretic peptides (NPs) has been demonstrated in experiments using adrenocortical cells from various animal species. 3′,5′-cyclic guanosine monophosphate (cGMP), which is produced by atrial natriuretic peptide (ANP), is recognized as a suppressor of aldosterone production. However, it has also been reported that cGMP does not exhibit the same aldosterone-suppressing effect as ANP. Therefore, we examined the effect of ANP on aldosterone suppression using various inhibitors and agonists, including a cGMP agonist.

**Design:**

We investigated the expression of ANP receptors in human adrenocortical cells. Next, we examined aldosterone synthesis gene expressions and the effects of various inhibitors, agonists, and siRNAs for the two ANP receptors on the aldosterone-suppressing action of ANP using human adrenocortical H295R cells, precultured with Ang-II. We also investigated inhibitory G (Gi) protein signaling as a cGMP-independent factor.

**Results:**

The human adrenocortical cell line H295R was shown to express the guanylyl cyclase A receptor (NPR1). Treatment with a neprilysin inhibitor ameliorated ANP-induced suppression of aldosterone production. Aldosterone production elevated by cyclic adenosine monophosphate (cAMP) agonist was significantly reduced by inhibitors of cAMP signaling. Conversely, cGMP alone had no significant aldosterone-suppressing effect. By suppressing the expression of receptors NPR1 and guanylyl cyclase C receptor (NPR3), aldosterone production increased in the presence of ANP. The suppression of aldosterone production by ANP was abolished by blocking Gi signaling by pertussis toxin (PTX).

**Conclusion:**

These results suggest that ANP suppresses aldosterone production in H295R cells through NPR1 and NPR3, and this suppression is partially mediated by a cGMP-independent Gi signaling.

## Background

Drug therapy for heart failure has shifted from diuretics and cardiac stimulants to treatments that suppress neurohumoral factors. The PARAMOUNT trial conducted in patients with HFpEF reported that the neprilysin inhibitor LCZ696 resulted in a greater reduction in NT-proBNP (N-terminal pro-B-type natriuretic peptide) compared to the valsartan group (1). In the PARADIGM-HF trial, which targeted patients with chronic heart failure with NYHA (New York Heart Association) class II or higher and a LVEF of 40% or less, the primary endpoint of cardiovascular death or hospitalization due to heart failure was significantly lower with sacubitril/valsartan than with enalapril (2).

Natriuretic peptides (NPs) consist of three ligands (ANP, BNP, and CNP). ANP is produced in the atria, and BNP is produced mainly in the ventricle. CNP is produced not only in vascular endothelial cells but also in bone tissue (3). They can bind to three different receptors: NPR1, guanylyl cyclase B receptor (NPR2), and NPR3, which are classified into two receptor types. NPR1 and NPR2 are guanylyl cyclase (GC) receptors, which use cyclic GMP as a second messenger, and NPR3 acts as a clearance receptor, which takes up NPs into cells and breaks them down. ANP has the highest binding affinity to clearance receptors, followed by CNP and BNP.

NP loses its activity when cleaved by a neutral endopeptidase called neprilysin.

Neprilysin is a neutral and a membrane-bound endopeptidase that is widely distributed in the body. Although clearance receptors are also involved in the degradation of diuretic peptides, the main degradation is by neprilysin, and inhibition of neprilysin increases endogenous diuretic peptides (4).

It has been reported that the aldosterone suppression effect of ANP is mediated by protein kinase G (PKG) (5) or phosphodiesterase 2 (PDE2) (6), which are activated by cGMP. However, cGMP alone did not exert an inhibitory effect in our study; we investigated the role of Gi, which inhibits adenylyl cyclase (AC) activity, as a cGMP-independent factor. Since PTX, which inhibits Gi, abolished the aldosterone suppression by ANP, we considered Gi to play a role in the suppressive effect of ANP.

This study suggested that the aldosterone-suppressing mechanism of ANP is mediated not only by NPR1 but also by NPR3, is partly cGMP-independent, and reduces AC activity, including inhibitory G proteins.

## Materials and methods

### Materials

NCI-H295R (H295R cells, CRL-110296) was purchased from American Type Culture Collection (Manassas, VA, USA). Cells were cultured and maintained in DMEM/Ham’s F-12 (Nacalai Tesque, Kyoto, Japan) containing 10% FBS (Sigma-Aldrich, St. Louis MO, USA) with 1% ITS-X (Invitrogen) and 1% penicillin and streptomycin (Nacalai Tesque, Kyoto, Japan). The following reagents and antibodies were used: ANP (4135, Peptide Institute, Osaka, Japan), angiotensin-II (4001, Peptide Institute, Osaka, Japan), ACTH (4109, Peptide Institute, Osaka, Japan), KCl (Wako, Tokyo, Japan), anti-NPR1 (55116_1, Proteintech, IL, USA), anti-GAPDH (10494_1, Proteintech, IL, USA), LBQ657 (Cayman chemical company, Ann Arbor, ML, USA), 8-bromo-cGMP (Tocris, Bristol, UK), forskolin (Sigma-Aldrich, St Louis, MO, USA), KT5823 (Almone Lab., Jerusalem, Israel), H-89 (LKT Lab., MN, USA), KH7 (Cayman chemical company, Ann Arbor, ML, USA), and PTX (Wako, Osaka, Japan). Fluo-8 AM (AAT Bioquest, CA, USA) and pluronic acid F-127 (AAT Bioquest, CA, USA) were used to measure intracellular Ca^2+^ levels.

### Immunoprecipitation and immunoblotting

NPR1 was immunoprecipitated using Sepharose G magnetic beads (Cytiva, Massachusetts, USA) according to the manufacturer’s procedures. Immunoblotting was performed by standard tank methods using Mini-PROTEAN TGX precast gel (Any-KD, Bio-Rad, Hercules, CA, USA) and PVDF membrane (GE Healthcare, Chicago, USA). Signals were detected using the appropriate horseradish peroxidase-conjugated secondary antibody (anti-rabbit IgG-HRP, Bio-Rad, 1706515; anti-mouse IgG-HRP, Proteintech, SA00001-1) and Chemi-Lumi One ECL detection kit (Nacalai Tesque Inc., Kyoto, Japan).

### Measurement of aldosterone synthesis

Human adrenocortical cells (H295R) were seeded in 24-well plates and grown to semi-confluence and then replaced to serum-free medium (0.2% ITS and 1% PS) with 10^−8^M Ang-II for 24 h. The cells were washed once with serum-free medium, and then, the serum-free medium was replaced with or without various compounds including ANP, and then, culture supernatants were collected after 24 h. In this study, to avoid complicating the experimental system by inhibiting the degradation of not only ANP but also Ang-II with LBQ657, which inhibits peptide degradation, we adopted a method to enhance aldosterone production capacity by Ang-II treatment before ANP administration. Thus, pre-treatment with Ang-II before administration of reagents is referred to as Ang-II pre-treatment (Pre Ang-II). The assessment of aldosterone levels in the cell culture medium was conducted in triplicate using an enzyme immunoassay kit (Abnova, Taoyuan, Taiwan). Before incubation with ANP, cells were incubated for 30 min to 1 h in serum-free medium containing LBQ657 and then replaced with new medium containing LBQ657 and ANP. Forskolin or 8-bromo-cAMP was used to examine the inhibitory effect of ANP on the increase in aldosterone induced by cAMP with or without Ang-II treatment. H89, a protein kinase A (PKA) inhibitor, or KH7, a selective soluble AC inhibitor, was added in the same way as ANP. Results are mean ± s.d. (*n* = 3). The asterisk denotes significant differences (*P* < 0.05) from the control group.

To investigate the effect of ANP on potassium- and ACTH-mediated aldosterone production, cells were precultured for 24 h in the presence of potassium (20 mM) or ACTH (100 nM), and the stimulus was removed before ANP administration (pre-K^+^ or pre-ACTH). On the other hand, ANP was administered simultaneously with potassium or ACTH. After 24 h, supernatants were collected and used for ELISA. Neprilysin inhibitor LBQ657 treatment was performed on all cells treated with ANP.

### Reverse transcriptase PCR analysis

Total RNA was extracted from H295R cells using an RNA extraction kit (Favorgen, PingTung, Taiwan). A spectrophotometer Implen NP-80 (Implen, Munich, Germany) was used to quantify RNA yields. cDNA was synthesized from total RNA (0.5–2μg) in a 10 μl volume using a high-fidelity RT kit (Applied Biosystems, Waltham, MA, USA). Real-time PCR was performed with QuantStudio 3 (Applied Biosystems, Waltham, MA, USA) using PrimeTime Gene expression Mix (IDT, Coralville, Iowa, USA). The thermal profile was comparative Ct fast. TaqMan primers used in this experiment were as follows: GAPDH (Hs.99999905_m1), NPR1 (Hs.01099745_m1), and NPR3 (Hs.01099013_m1). For the expression analysis of the following aldosterone synthesis genes, the retrieval time was decided as previously reported (7): NR4A2 (Hs.01117527_g1), CYP11A1 (Hs.00167984_m1), CYP11B2 (Hs.01597732_m1), HSD3B2 (Hs.00605123_m1), and StAR (Hs.00986559_g1). Amplification of the sequence of interest was normalized with reference endogenous gene GAPDH. The value was expressed as a ratio to control. Each sample was quantified against the housekeeping gene GAPDH and normalized to the control group.

### siRNA experiments

siNPR1 and siNPR3 were purchased from Integrated DNA Technologies (Coralville, IA, USA). In this study, we used two siRNAs with different sequences: hs.Ri.NPR1.13.2 (siNPR1#1) and hs.Ri.NPR1.13.3 (siNPR1#2) for siNPR1 or hs.Ri.NPR3.13.2 (siNPR3#1) and hs.Ri.NPR3.13.3 (siNPR3#2) for siNPR3. DS NC1, siNPR1, and siNPR3 siRNAs were transfected at the time of plating using Lipofectamine RNAi MAX (Thermo Fisher Scientific, MA, USA). The cells were harvested for qRT-PCR after 4–5 days of siRNA transfection.

### Cytosolic calcium measurement in adrenocortical cells

Cells were incubated confluently in 96-well plates. After replacing the serum-free medium, cells were loaded with 5 μM Fluo-8 AM diluted in HBSS (+) buffer (Nacalai Tesque, Kyoto, Japan) with 0.02% pluronic acid F-127 for 1 h at 37°C and washed two times with HBSS (+). Images were acquired every two seconds with a Nikon Ti-E2 microscope (Nikon, Tokyo, Japan) equipped with STX-APP (Tokai Hit, Shizuoka, Japan), and the software used for the acquisitions was NIS Element AR (Nikon). Exposure time was set to 75 ms. Changes in Fluo-8 were expressed as F/F0, where F0 is the average of fluorescent intensity before Ang-II or buffer administration.

### Statistical analysis

The results are expressed as the mean ± s.d. of three or more determinations. The result in Fig. 2A is representative of four independent experiments. The result in Fig. 5A is representative of five independent experiments. All tests were conducted with *n* = 3. The significance of differences among measured values was evaluated with analysis of variance using paired Student’s t-test. Statistical significance was set at < 0.05.

## Results

### Expression of NPR1 in human adrenocortical cell lines

**Figure 1:**
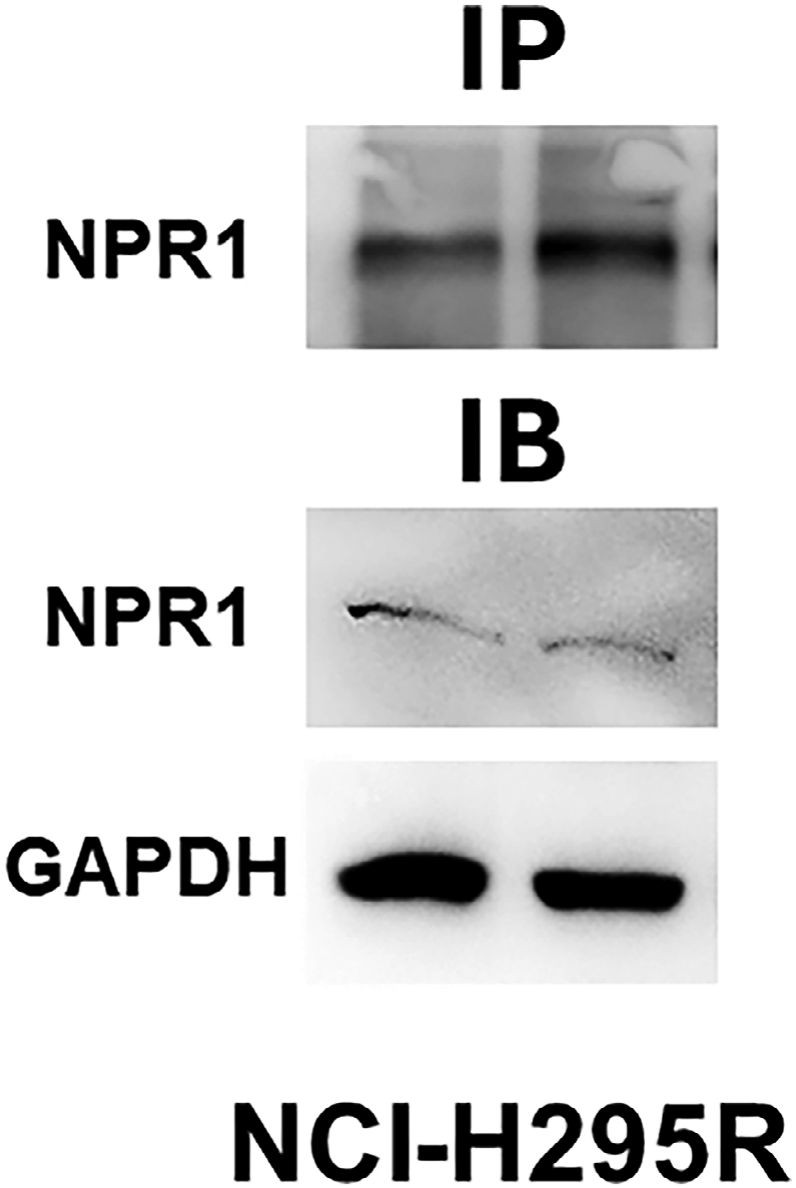
Expression of NPR1 in human adrenocortical cells. Protein expression of NPR1 by immunoprecipitation and immunoblotting in human adrenocortical cells.

NPR1 expression was confirmed in human adrenocortical cells, but its expression was considerably weak (Fig. 1).

### ANP-mediated attenuation of aldosterone production from H295R cells

**Figure 2:**
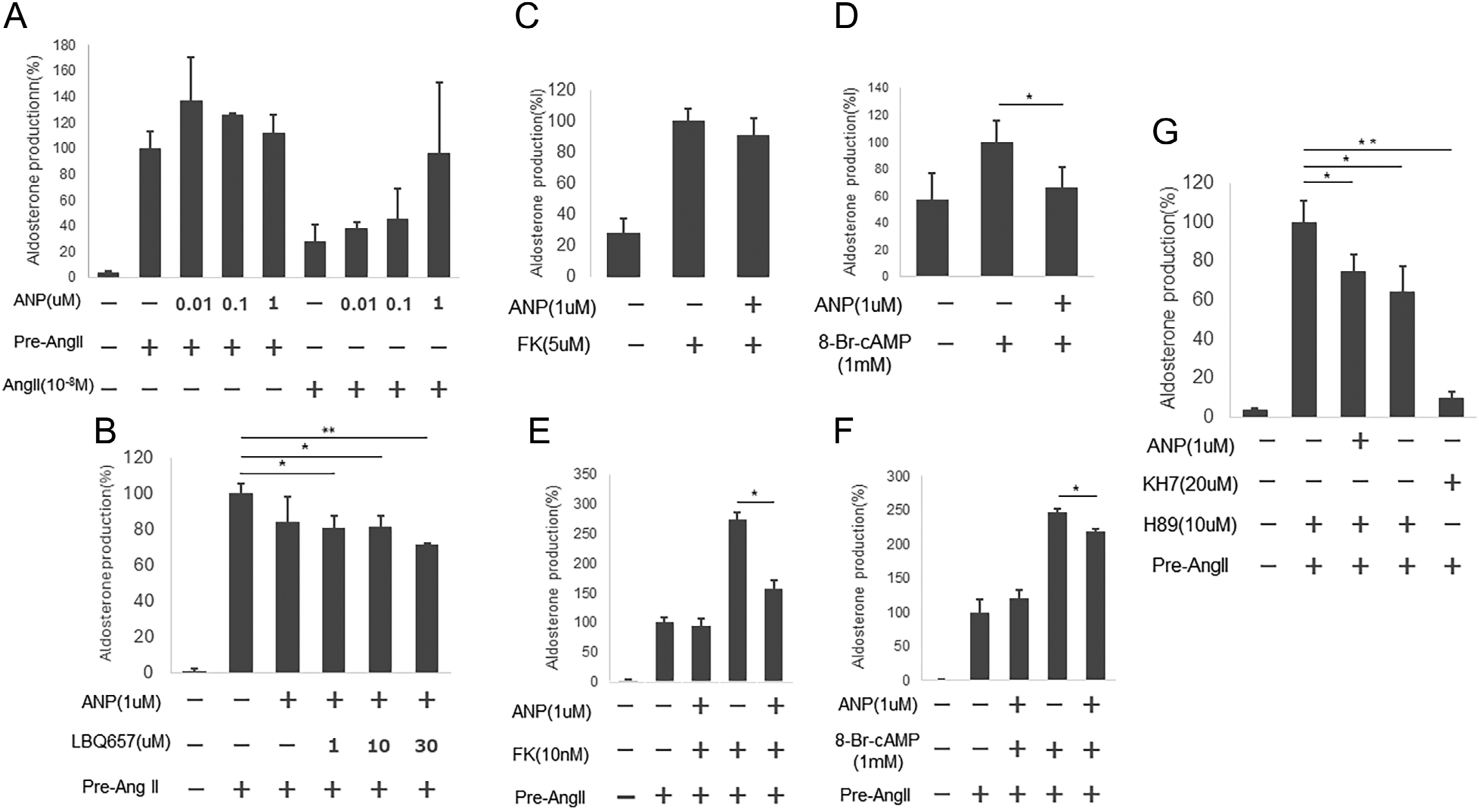
Aldosterone production from H295R cells. (A) Aldosterone production from H295R cells in the presence of ANP (0.01, 0.1, 1 μM). (B) Aldosterone production from H295R cells in the presence of various concentrations of LBQ657 (1, 10, 30 μM). (C) Aldosterone production from H295R cells in the presence of forskolin (5 μM) or (D) 8-Br-cAMP (1 mM). (E) Aldosterone production from H295R cells pretreated with Ang-II and then incubated in the presence of forskolin (1 μM) or (F) 8-Br-cAMP (1 mM), and (G) KH7 (10 μM) and H89 (20 μM). Cells were pretreated with Ang-II (10^−8^M) before the addition of indicated compounds including ANP, except for (C) or (D). Results are mean of s.d. Significant differences: *P < 0.05, **P < 0.01 from control values.

Since the suppressing effect of ANP alone on aldosterone production was inconsistent (Fig. 2A), the neprilysin inhibitor LBQ657 was added between 30 min and one hour before ANP. Pretreatment of LBQ657 showed significant suppressing effects in H295R cells (Fig. 2B). Furthermore, by treating cells with Ang-II for 24 h, it is possible to obtain cells capable of producing aldosterone, and there is no need to consider the effects of neprilysin inhibitor. To examine the suppressive effect of ANP on cAMP-mediated signaling in aldosterone production, forskolin, an activator of AC producing cAMP, and the 8-bromo-cAMP agonist were used under two conditions: untreated (Fig. 2C and D) and pretreatment with Ang-II (Fig. 2E and F). Except for the cells treated with forskolin alone, ANP showed significant suppression of aldosterone production. Moreover, blocking of cAMP production by KH7, a soluble AC-specific inhibitor, or H89, a selective inhibitor of cAMP-dependent protein kinase, was significantly suppressed on aldosterone production from H295R cells pretreated with Ang-II (Fig. 2E). These results suggest that the inhibitory effect of ANP is dependent on the decrease in cAMP concentration. Both high potassium levels and ACTH induced aldosterone production, but no significant inhibitory effect was observed in the presence of ANP (Supplementary Fig. 2A and B (see section on Supplementary materials given at the end of the article)). In addition, cells precultured 24 h prior to the administration of ANP showed stronger aldosterone production compared to untreated cells. A slight tendency to reduce aldosterone production was observed in ACTH-pretreated cells in the presence of ANP, but there was no statistical significance (Supplementary Fig. 2B).

### Gene expression related to aldosterone synthesis

**Figure 3:**
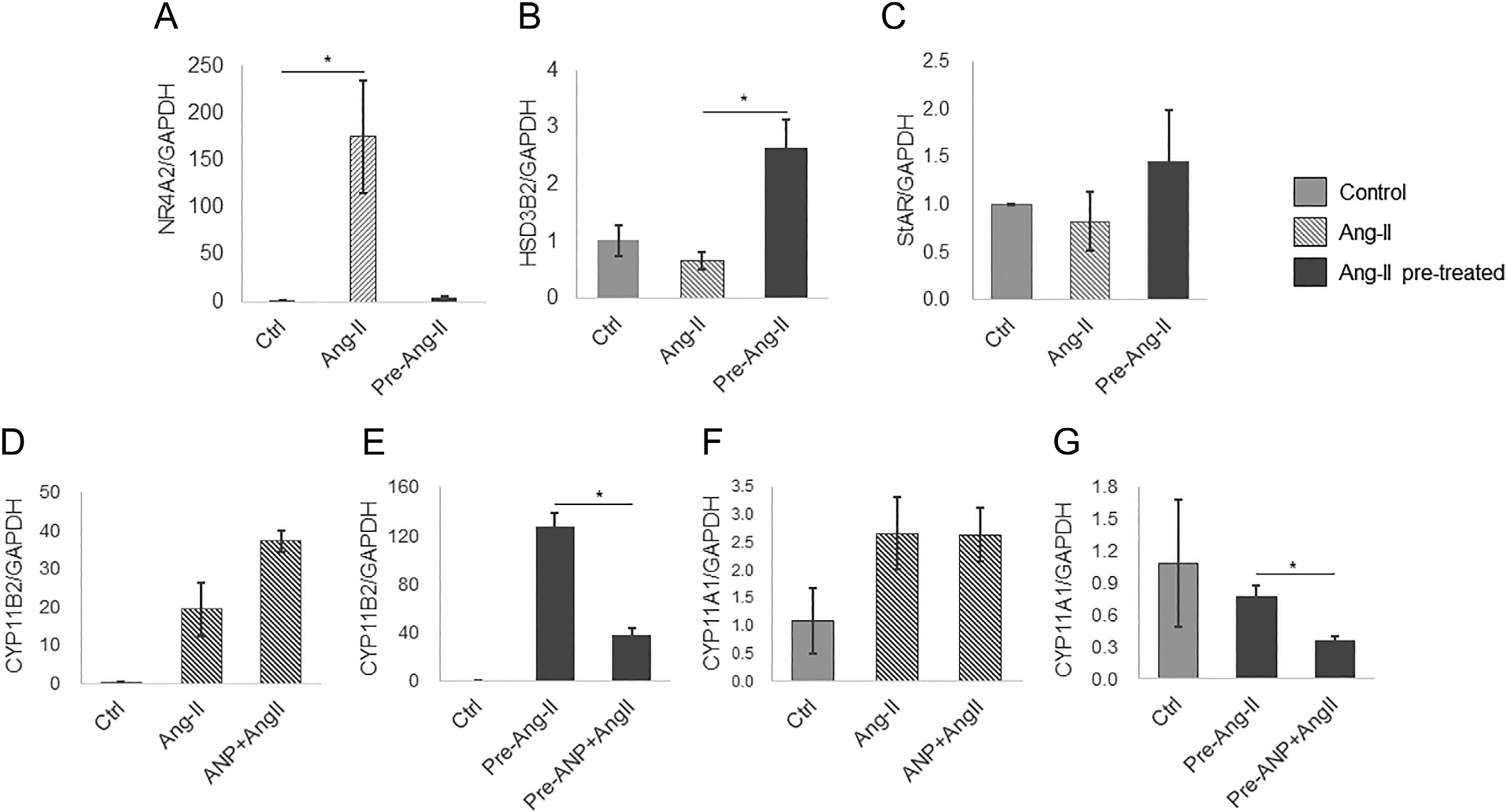
Gene expressions related to aldosterone synthesis in H295R cells. Aldosterone synthesis gene expressions in H295R cells were measured under two conditions. Cells were treated either with Ang-II or in serum-free medium after pretreatment with Ang-II for 3 h. Cells were collected and used for gene expression analysis. Gene expression in Ang-II-treated and Ang-II-pretreated cells in the presence or absence of ANP. (A) NR4A2, (B) HSD3B2, (C) StAR, (D and E) CYP11B2, and (F and G) CYP11A1. Results are mean of s.d. Significant differences: *P < 0.05, **P < 0.01, versus control, *n* = 3.

Next, the expression of genes involved in aldosterone synthesis was measured under two conditions: one condition was three hours after Ang-II administration and the other was three hours after Ang-II removal following pretreatment with Ang-II for 24 h. NR4A2 (Fig. 3A), HSD3B2 (Fig. 3B), StAR (Fig. 3C), CYP11B2 (Fig. 3D and E), and CYP11A1 (Fig. 3F and G) showed significantly elevated expression compared to the control under both conditions. Notably, CYP11B2 and CYP11A1 showed significant decreases in expression by ANP addition in cells pretreated with Ang-II for 24 h.

### Effects of siNPR1 or siNPR3 on aldosterone production from H295R cells

**Figure 4:**
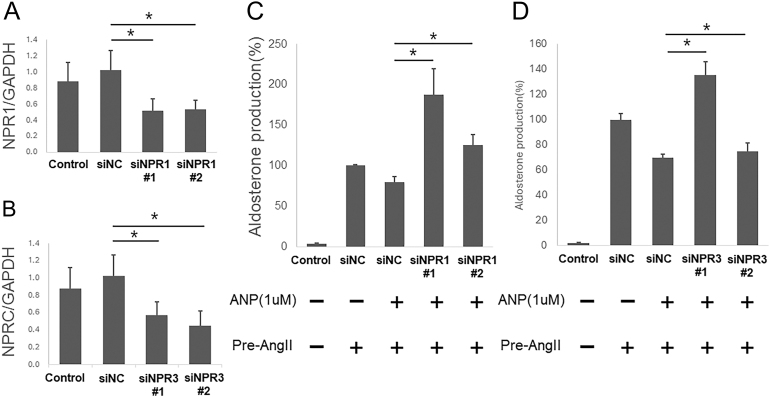
Effects of NPR1 and NPR3 on aldosterone production from H295R cells. Aldosterone production from H295R cells transfected with siNPR1 or siNPR3. (A) NPR1 or (B) NPR3 mRNA expression. The RNA used for RT-qPCR was extracted from the cell cultured four to five days after siRNA transfection (*n* = 3). NPR1 or NPR3 mRNA was normalized by GAPDH mRNA (C and D). Aldosterone production after the transfection of siRNA. Cells were pretreated with Ang-Ⅱ (10^−8^M) before the addition of ANP. Results are mean of s.d.; significant differences: *P < 0.05, **P < 0.01 from values in siRNA-negative control, *n* = 3. The results are represented as fold increase ± s.d.

Since ANP binds to two receptors, NPR1 and NPR3, we suppressed the expression of NPR1 and NPR3, respectively, using two siRNAs targeting different sequences of mRNA. Both receptor expression and suppression were examined by reverse transcription and real-time quantitative PCR (Fig. 4A and B). Aldosterone production from the cells that had incorporated siRNA was significantly increased in the presence of ANP compared to the negative control (Fig. 4C and D). Thus, it was suggested that aldosterone suppression by ANP is mediated through both NPR1 and NPR3.

### Role of inhibitory G-protein signaling on aldosterone production

**Figure 5:**
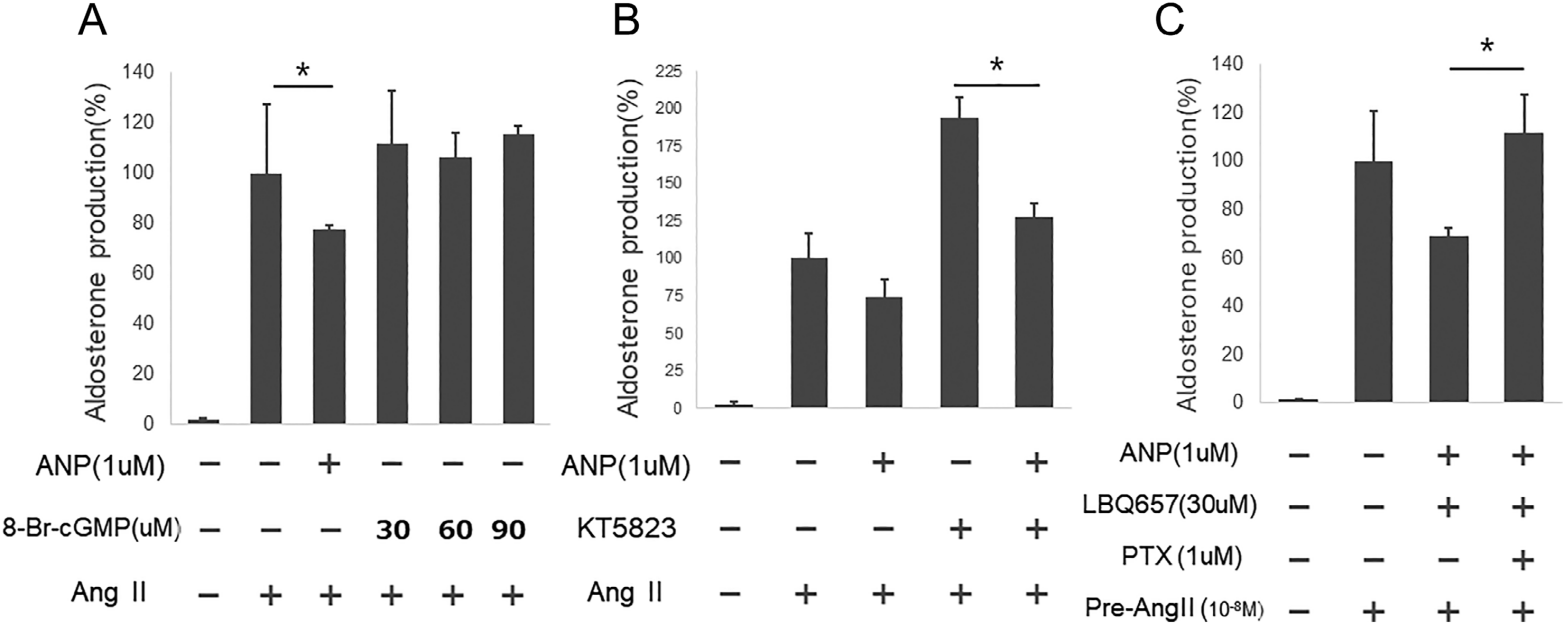
Role of inhibitory G signaling in aldosterone production in H295R cells. (A) Aldosterone production from H295R cells in the presence of a cGMP agonist, 8-bromo-cGMP, (B) in the presence of a PKG inhibitor, KT5823. Cells were pretreated with Ang-II before the addition of cGMP or ANP. (C) Aldosterone production from H295R cells with or without PTX (1 μg/mL). Results are mean of s.d.; significant differences: *P < 0.05, **P < 0.01, versus control, *n* = 3.

To demonstrate that effects of cGMP analog, 8-bromo-cGMP was used on aldosterone suppression. Various concentrations of cGMP did not have a significant inhibitory effect in H295R cells (Fig. 5A). Moreover, the inhibitory effect of ANP remained with the treatment of KT5823, the inhibitor of cGMP-dependent protein kinase (Fig. 5B). These results suggest that cGMP-mediated inhibition does not work in this experimental system using adrenocortical cells. In our experimental condition, treatment of cGMP inhibitor did not have any influence on aldosterone production in H295R cells. Therefore, next, we examined the effect of inhibitory G protein. Activation of a Gi protein inhibits AC activity and produces lower intracellular cAMP. H295R cells were incubated with or without PTX, widely used for inhibition of Gi signaling. The suppressive effect of ANP was significantly canceled in the presence of PTX (Fig. 5C). These results suggest that ANP suppresses aldosterone partially through a cGMP-independent pathway.

## Discussion

It has been observed that ANP, produced in the atria, markedly reduces aldosterone synthesis triggered by angiotensin II, low potassium, cAMP agonists, and ACTH within adrenocortical glomerulosa cells isolated from rats, cows, and human adrenal tumors (8). ANP receptor expression has been observed in humans through northern blotting (9) or immunohistochemistry (10). In rats, the expression and function of not only NPR1 but also NPR2 and NPR3 have been mentioned (11). However, most of ANP and BNP are degraded by the peptide-degrading enzyme neprilysin, which is widely present in the body. Studies utilizing human adrenal cortical cells revealed that ANP with an altered neprilysin degradation locus demonstrated a substantial capacity to inhibit aldosterone (6). In this study, we first examined the suppressive effect of ANP on aldosterone production using adrenocortical cells pre-incubated with Ang-II; however, adjusting the concentration and incubation time, no reproducible inhibitory effect was obtained. In the presence of the neprilysin inhibitor LBQ657, significant aldosterone suppression was observed in the presence of ANP. Neprilysin plays a critical role in the regulation of physiological processes by degrading various bioactivity peptides and has been identified to degrade not only ANP but also peptides, such as Ang-I and Ang-II as substrates (12); therefore, in the presence of a neprilysin inhibitor, Ang-II accumulates. In this study, we were able to investigate the effects of ANP without considering the effect of neprilysin inhibitors on Ang-II by first preculturing the cells with Ang-II alone and then removing it.

We also investigated the effect of ANP on high potassium- and ACTH-induced aldosterone elevation, but neither showed any suppression by ANP (Supplementary Fig. 2A and B). H295R cells are known to have low expression of the ACTH receptor MC2R (13, 14); therefore, aldosterone production in response to ACTH has been reported to be weak in H295R cells. In our observation, the cells treated with ACTH showed relatively high aldosterone production compared to untreated cells. To investigate the effect of ANP on intracellular Ca^2+^ concentration, we measured the changes in intracellular Ca^2+^ using Ang-II-pretreated cells, but there were no significant changes in the presence of ANP. One possible reason is that Ca^2+^ concentration was already elevated by Ang-II treatment, so the effect of ANP on intracellular Ca^2+^ concentration is considered to be undetectable (Supplementary Fig. 3).

Next, we investigated which receptors are important in ANP signal transduction. ANP binds to two receptors, NPR1 and NPR3. In our experiments using siRNA, the suppression of aldosterone by ANP was relieved by both siNPR1 and siNPR3 compared to negative controls, suggesting that ANP acts via both cGMP-dependent and cGMP-independent pathways. The ANP receptor NPR1 is coupled to the guanylate cyclase/cGMP system, while NPR3 is primarily involved in the degradation of NPs and has a different structure from NPR1 in its cytoplasmic domain (15). The NPR3 receptor has a single transmembrane domain and a short cytoplasmic domain of 37 amino acids, and its structural specificity and amino acid sequences activates Gi regulatory protein and associated signaling (16). Stimulation of NPR3 inhibits AC activity via Gi signaling in A10 vascular smooth muscle cells (17, 18) and other cells, including the adrenal cortex (19). In contrast, experiments using CNP agonists have not shown any aldosterone-inhibiting effect (6), and aldosterone levels or blood pressure responses in human remained unchanged by CNP administration (20).

We also measured cytochrome P450 family 11 subfamily b member 2 (CYP11B2), an index of aldosterone synthase in siRNA experiments, but no changes in CYP11B2 mRNA were found to correlate with aldosterone concentrations. The increase in CYP11B2 mRNA peaks soon after Ang-II administration (6–8 h later, unpublished data), and therefore, in the experimental conditions of this study, approximately 48 h had passed since Ang-II administration, so it is thought that no significant change in CYP111B2 was observed. However, significant increases were observed shortly after both the administration of Ang-II and the removal of Ang-II for the following genes: NR4A2, CYP11A1, HSD3B2, StAR, and CYP11B2. NR4A2 expression in cells treated with Ang-II was significantly increased compared to expression in cells precultured with Ang-II (3 h after the removal of Ang-II). ANP treatment significantly reduced the expression of both CYP11B2 and CYP11A1 in Ang-II-pretreated cells, suggesting that ANP is also involved in gene expression related to aldosterone synthesis. The possible reason for the decreased expression of CYP11B2 and CYP11A1 by ANP in cells pretreated with Ang-II but not in untreated cells is that Ang-II treatment may have caused either (i) increased ANP receptor expression in H295R cells or (ii) an influence on gene expression of CYP11B2 and CYP11A1, allowing for the detection of ANP effect, or both.

In our experiments, we did not observe any aldosterone-inhibiting effect of cGMP. However, since aldosterone production also increase when NPR1 was blocked, it is suggested that cGMP is involved in aldosterone suppression of ANP.

In our experiments, we did not observe any inhibitory effect from cGMP alone. However, PDE2, one of the PDEs activated by cGMP (21, 22), was highly expressed in the adrenal cortex (18), and PDE2 activated by cGMP elevated by ANP rapidly reduced intracellular cAMP concentration (22). Furthermore, H295R with suppressed PDE2 expression showed increased aldosterone production and CYP11B2 expression (6). On the other hand, permeable cGMP analogs failed to inhibit basal or stimulated aldosterone production using animal adrenal cells and also showed that cGMP increased aldosterone production (8, 23, 24). It has been reported that aldosterone production increased due to cGMP-induced protein kinase II (PKGII) over expression (24). It is hypothesized that there is some difference between cGMP administered alone and cGMP produced by ANP.

Interestingly, H295R cells have little-to-no expression of PKGII compared to adrenocortical cells of rats fed with a low-sodium diet (24); our experiments showed that H295R cells treated with Ang-II had slightly increased aldosterone levels in the presence of cGMP. Ang-II treatment may have increased PKGII expression in H295R cells. Therefore, the inhibitory effect of ANP is maintained even in the presence of a PKG inhibitor, activated by cGMP, suggesting the existence of a PKG-independent aldosterone inhibitory pathway.

Previous reports have suggested that CNP is involved in the activation of Gi signaling (16, 19, 20). As a cGMP-independent factor in the aldosterone suppression by ANP, Gi-signaling was examined. PTX, a selective inhibitor of Gi signaling, significantly abolished the aldosterone suppression of ANP. The result indicated that Gi signaling is involved in the aldosterone suppression of ANP. Gi is one of the heterotrimeric G protein alpha subunits that, when activated, inhibit AC, leading to a decrease in the intracellular concentration of cAMP.

We also investigated the expression of RGS4 as a factor in a cGMP-independent manner in the aldosterone suppression mechanism of ANP.

RGS4 mRNA has been detected in the adrenal cortex, and RGS4 protein, which protects cardiac health, is also found in heart muscle. An RGS4 inhibitor eliminates the suppressive effect of ANP on aldosterone production (5, 25). In diencephalic glial cells, ANP and cGMP cause RGS4 to move from the cytosol to the plasma membrane (26), and increased RGS4 expression reduces aldosterone production in H295R cells (27). In preliminary experiments, we also examined the expression of RGS4. Unlike glial cells, or aldosterone-producing cells (APA), we did not detect RGS4 protein expression in H295R cells; however, there was a significant increase in mRNA expression in a short period of time with the addition of Ang-II. Despite the hypothesis that ANP influences RGS4 action in adrenal cortical cells, immunofluorescent results showed no clear membrane translocation or changes in RNA expression with ANP.

**Figure 6:**
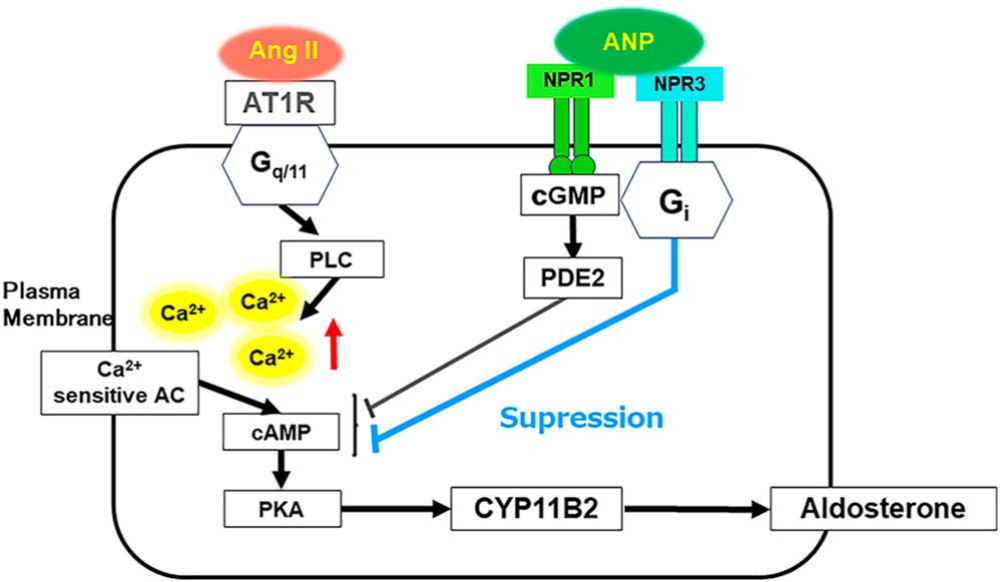
Proposed mechanism of ANP suppression of aldosterone production stimulated by Ang-II. A schematic diagram illustrating the inhibition of aldosterone production by ANP in adrenocortical cells. Angiotensin II induces aldosterone production through an increase in cytoplasmic Ca^2+^ concentration and further activation of Ca^2+^-sensitive adenylyl cyclase (AC). On the other hand, ANP binds to NPR1 and NPR3, increasing cGMP and activating Gi signaling, thereby decreasing cAMP and inhibiting aldosterone synthesis.

Figure 6 shows a hypothetical scheme of how ANP suppresses aldosterone production in adrenocortical cells. Ang-II binds to the G protein-coupled receptor α_q/11_ (G_q/11_), coupled with angiotensin type 1 receptor (AT1R) and activates phospholipase C (PLC), which increases Ca^2+^, followed by an increase in cAMP through Ca^2+^-sensitive AC (28, 29), and induces aldosterone production. ANP binds to NPR1, increasing cGMP, which then binds to PDE2, whereas it activates Gi signaling while binding to NPR3. The combined action of these receptor bindings reduces cAMP and inhibits aldosterone production. In summary, this study demonstrates that ANP inhibits aldosterone production by decreasing cAMP concentrations and, to a certain extent, through a cGMP-independent mechanism involving NPR1 and NPR3.

## Supplementary materials







## Declaration of interest

The authors have no commercial or financial involvement related to this research that constitutes or may be perceived as a conflict of interest.

## Funding

This research was supported by a Grant-in-Aid for Scientific Research from the Japan Society for the Promotion of Science (JSPS KAKENHI, Grant Number: 23K08788).

## Author note

Due to the exclusion of human subjects and animal experimentation, this study did not necessitate formal ethical approval.
