# Supplementary material for: Inhibitory mechanism of ANP on aldosterone production in human adrenocortical cells
Source: Endocr Connect. 2026 Aug 12;15(8):e260107. doi: 10.1530/EC-26-0107 (PMC13482795; doi:10.1530/EC-26-0107)
Supplement: Supplementary file 1 [file EC-26-0107_supplementary_figure_1.pdf]

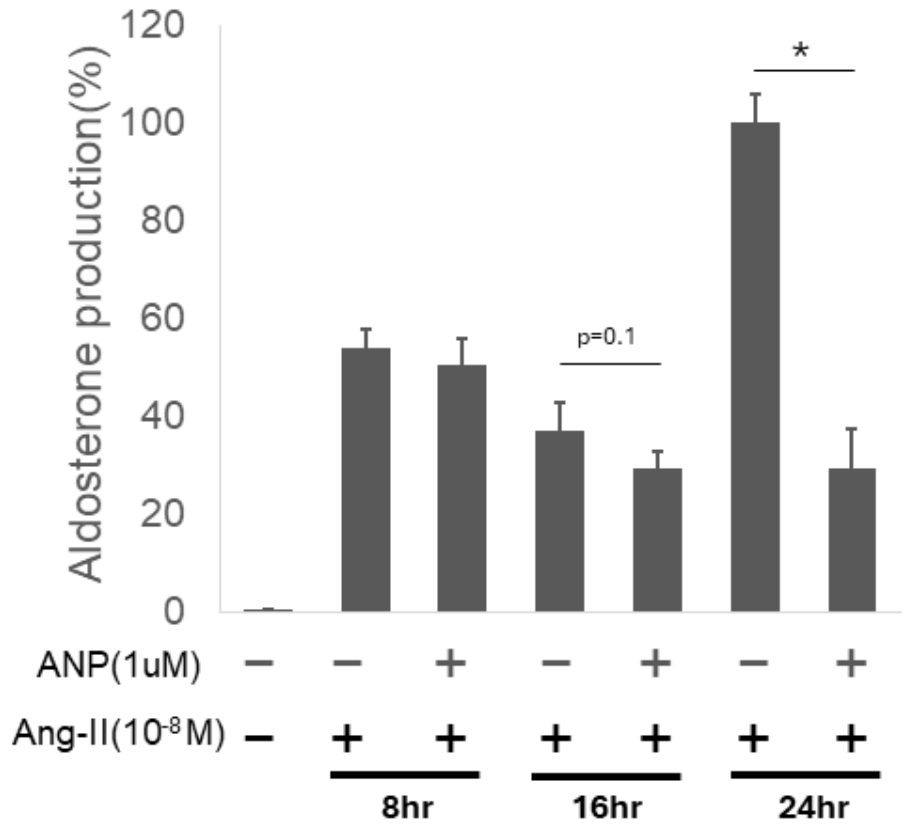

Aldosterone production(%)
120
100
80
60
40
20
0
p=0.1
*
ANP(1uM)
Ang-II(10^{-8}M)
8hr
16hr
24hr
