# Supplementary material for: Inhibitory mechanism of ANP on aldosterone production in human adrenocortical cells
Source: Endocr Connect. 2026 Aug 12;15(8):e260107. doi: 10.1530/EC-26-0107 (PMC13482795; doi:10.1530/EC-26-0107)
Supplement: Supplementary file 2 [file EC-26-0107_supplementary_figure_2.pdf]

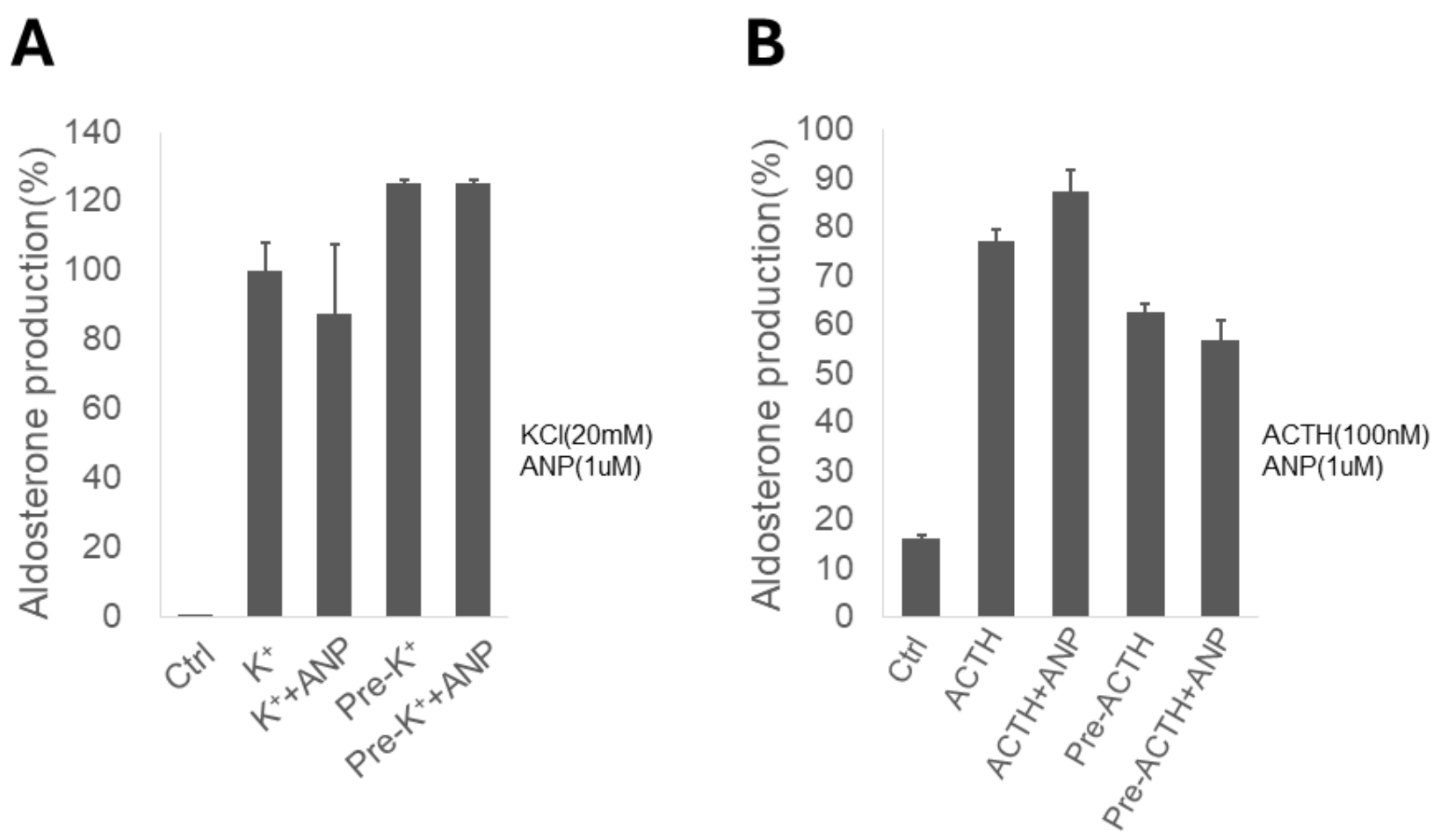

A
Aldosterone production(%)
140
120
100
80
60
40
20
0
Ctrl
K+
K+ +ANP
Pre-K+
Pre-K+ +ANP
KCl(20mM)
ANP(1uM)
B
Aldosterone production(%)
100
90
80
70
60
50
40
30
20
10
0
Ctrl
ACTH
ACTH+ANP
Pre-ACTH
Pre-ACTH+ANP
ACTH(100nM)
ANP(1uM)
