# Supplementary material for: Inhibitory mechanism of ANP on aldosterone production in human adrenocortical cells
Source: Endocr Connect. 2026 Aug 12;15(8):e260107. doi: 10.1530/EC-26-0107 (PMC13482795; doi:10.1530/EC-26-0107)
Supplement: Supplementary file 3 [file EC-26-0107_supplementary_figure_3.pdf]

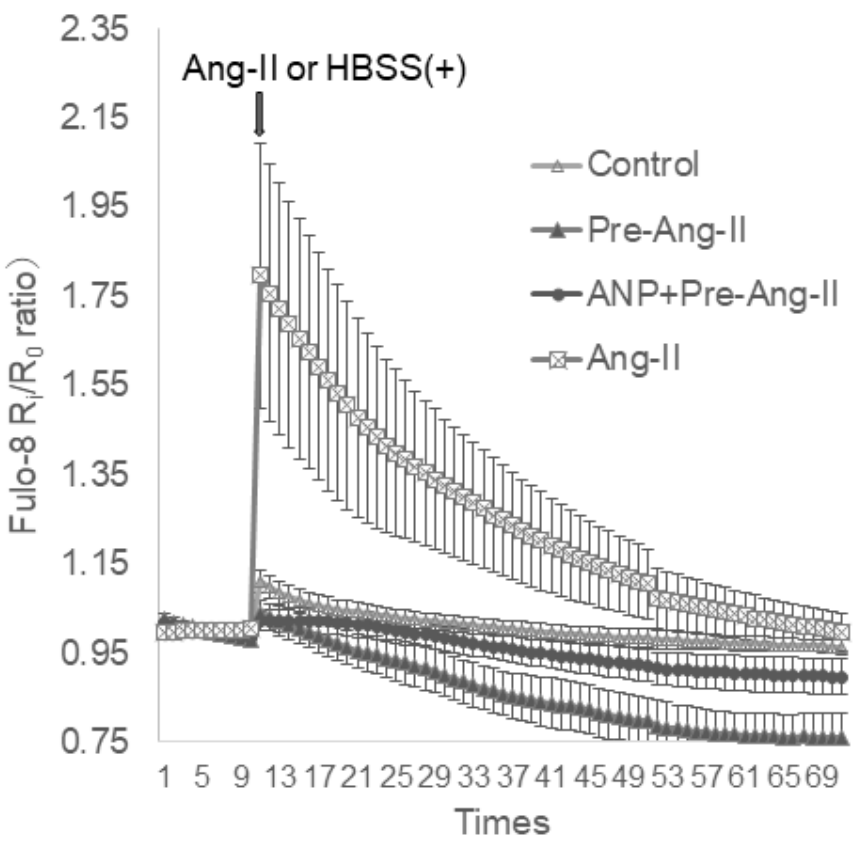

Ang-II or HBSS(+)
Control
Pre-Ang-II
ANP+Pre-Ang-II
Ang-II
2.35
2.15
1.95
1.75
1.55
1.35
1.15
0.95
0.75
1 5 9 13 17 21 25 29 33 37 41 45 49 53 57 61 65 69
Times
